# Fabrication and Properties of Tree-Branched Cellulose Nanofibers (CNFs) via Acid Hydrolysis Assisted with Pre-Disintegration Treatment

**DOI:** 10.3390/nano12122089

**Published:** 2022-06-17

**Authors:** Jun Li, Dongyan Liu, Junsheng Li, Fei Yang, Guoxin Sui, Yu Dong

**Affiliations:** 1 Shi-changxu Innovation Center for Advanced Materials, Institute of Metal Research, Chinese Academy of Sciences, Shenyang 110016, China; jli19b@imr.ac.cn (J.L.); fyang20s@imr.ac.cn (F.Y.); gxsui@imr.ac.cn (G.S.); 2 School of Materials Science and Engineering, University of Science and Technology of China, Shenyang 110016, China; 3 Engineering Center of National New Raw Material Base Construction of Liaoning Province, Shenyang 110031, China; lijunsheng_21@163.com; 4 School of Civil and Mechanical Engineering, Curtin University, P.O. Box U1987, Perth, WA 6845, Australia; y.dong@curtin.edu.au

**Keywords:** nanocellulose, tree-branched cellulose nanofibers (CNFs), acid hydrolysis, wood pulps, disintegration treatment

## Abstract

In this paper, the novel morphology of cellulose nanofibers (CNFs) with a unique tree-branched structure was discovered by using acid hydrolysis assisted with pre-disintegration treatment from wood pulps. For comparison, the pulps derived from both softwood and hardwood were utilized to extract nanocellulose in order to validate the feasibility of proposed material fabrication technique. The morphology, crystalline structures, chemical structures, and thermal stability of nanocellulose were characterized by means of transmission electron microscopy (TEM), X-ray diffraction (XRD) analysis, Fourier transform infrared spectroscopy (FTIR), as well as thermogravimetric analysis (TGA). Prior to acid hydrolysis, softwood and hardwood pulps underwent the disintegration treatment in the fiber dissociator. It has been found that nanocellulose derived from disintegrated pulps possesses much longer fiber length (approximately 5–6 μm) and more evident tree-branched structures along with lower degree of crystallinity when compared with those untreated counterparts. The maximum mass loss rate of CNFs takes place at the temperature level of approximately 225 °C, and appears to be higher than that of cellulose nanowhiskers (CNWs), which might be attributed to an induced impact of amorphous content. On the other hand, disintegration treatment is quite beneficial to the enhancement of tensile strength of nanocellulose films. This study elaborates a new route of material fabrication toward the development of well-tailored tree-branched CNFs in order to broaden the potential widespread applications of nanocellulose with diverse morphological structures.

## 1. Introduction

Cellulose is composed of 1, 4−β−glucopyranose units, which is the main component of most natural materials such as wood [1], cotton [2], flax [3], bacteria [4], and sea creatures [5]. It has active hydroxyl functional groups (−OH) located at C2, C3, and C6 of repeated unit glucose to contribute to the feasible functionalization for cellulose molecules [6]. Cellulose has been used as raw materials or additives in a variety of composite materials, reinforcing fillers [7], packaging films [8], adsorbents [9], dispersants [10], separator membranes [11], porous materials [12,13], etc.

More recently, nanocellulose has attracted more and more attention in academia and industry because of numerous advantageous features including large surface area, aspect ratio, high mechanical strength, renewability, as well as the abundance of raw materials in addition to biocompatibility and biodegradability [14]. 

The morphology, structure, size, and property of nanocellulose usually depend on material source and fabrication methods. Up till now, three types of nanocellulose have been widely manufactured, namely cellulose nanocrystals (CNCs) or cellulose nanowhiskers (CNWs), cellulose nanofibers (CNFs) or cellulose microfibrils/nanofibrils (CMFs/CNFs), and bacterial cellulose (BC) [15,16] leading to diverse micro/nanostructures. Generally, CNCs/CNWs are usually extracted by acid/enzymatic hydrolysis in rod-like or needle-like shape with a high degree of crystallinity [17,18]. CMFs/CNFs with specific lengths of several micrometers, on the other hand, exhibit much higher aspect ratios as opposed to CNCs/CNWs with a typical length of a few hundred nanometers. This is because amorphous phase is removed in CNCs/CNWs but remains in CNFs/CMFs with more flexibility [19]. Therefore, CNFs induce better reinforcing effects of composites with enhanced mechanical and excellent functional properties in relation to flocculability, dispersants, strong 3D-structures, and wound dressing carriers owing to their larger aspect ratios, as compared to CNCs/CNWs. In general, CNFs are obtained via high-speed shear and friction mechanism to disassemble the fibrils into nanofibers [20]. CNFs/CMFs are easily transformed into 3D disorganized networks due to the incomplete disintegration of cellulose fibers. Their sizes are associated with input energy and homogenization pass [21,22]. In particular, BC, exhibiting 3D networks randomly arranged by nanocellulose with high purity, is generally produced from acetobacter. In practice, it is regarded as promising biomedical materials especially in the field of wound healing and tissue engineering [23]. 

According to the previous literatures [17], rod-like or needle-like nanocellulose is usually achieved by using acid hydrolysis. In particular, the extraction of rod-like CNWs was the main focus with the length of several hundred nanometers from flax fibers and bacterial cellulose via sulfuric acid hydrolysis in our previous study [24]. Recently, we selected wood pulps as raw materials to extract nanocellulose. As the two processing methods used for the production of paper, disintegration and beating are always applied to loosen and delaminate the pulps, respectively. In order to explore the effect of pre-treatment on the extraction of nanocellulose, the mild treatment disintegration with less damage on pulp fibers was tried prior to acid hydrolysis in order to make a comparison with the undisintegrated pulps. Excitingly, nanocellulose extracted from wood pulps with novel tree-branched morphology was obtained. In particular, a more typical tree-branched characteristic becomes pronounced using pre-disintegration treatment prior to acid hydrolysis. We believe that the disintegration treatment favors the loosening and splitting of the microfibers, leading to the formation of nanocellulose with longer fiber length and more branches. It has been reported that tree-like cellulose nanofibrils can be produced by mechanically defibrillating refined cotton and wood pulps with the aid of a microfluidizer [25]. Accordingly, these modified cellulose nanofibrils in possession of tree-like formation have been proven to be excellent stabilizers for oil-based Pickering emulsions owing to the confinement effect of such unique 3D networks of tree-like structures. This study aims to holistically investigate morphology, micro/nanostructures, thermal and mechanical properties of tree-branched nanocellulose films extracted from a variety of wood pulps for potential development of ecofriendly thin film technologies utilized in advanced composite materials.

## 2. Experimental Work

### 2.1. Materials

Two commercial wood pulps, bleached softwood pulp sheets (Bratsk, Russia, BSP) and bleached hardwood pulp sheets (Bratsk, Russia, BHP) were received from the Engineering Center of National New Raw Material Base Construction of Liaoning Province, Shenyang, China. Softwood pulps were mainly produced from Korean pine, white pine, and larch. Hardwood pulps were derived from birch and poplar. The wood went through a series of pulping and bleaching to retain main cellulose in bleached pulps. The main chemical composition of BSP consists of 86.89 wt% α−cellulose, 9.72 wt% pentosan, and 0.18 wt% ash. Whereas BHP also comprises 80.72 wt% α−cellulose, 17.37 wt% pentosan, and 0.22 wt% ash. Sulfuric acid (H_2_SO_4_, AR, concentration: 98 wt%) was obtained from the Sinopharm Group Shenyang Co., Ltd., Shenyang, China, and sodium hydroxide (NaOH, AR, concentration: 95 wt%) was purchased from Shanghai Aladdin Reagent Co., Ltd (Shanghai China). Deionized (DI) water was used for solution preparation. All chemicals were used without further purification.

### 2.2. Methods

#### 2.2.1. Disintegration of Wood Pulps

The disintegration of wood pulps was carried out on a fiber dissociator (Dongguan International Material Tester Equipment Co. Ltd., Dongguan, China). BSP and BHP underwent disintegration treatment before acid hydrolysis, which were further compared with corresponding pulps without pre−disintegration treatment. A slurry of 2 wt% wood pulps was disintegrated at room temperature with a stirring speed of 1500 r/min for 20 min. The slurry of disintegrated fibers was subsequently filtered and dried in an air−circulating oven at 40 °C, which are referred to as BSP−D and BHP−D.

#### 2.2.2. Alkalization of Wood Pulps

Before the extraction of nanocellulose, all pulps were initially immersed into 5 wt% sodium hydroxide solution and then heated at 80 °C for 1 h via vigorous stirring at 1000 r/min. Subsequently, the pulp slurry was washed with DI water toward its neutrality. Finally, the slurry was filtered and dried in an air-circulating oven at 40 °C. This process was conducive to remove residual hemicellulose and further swell the pulps.

#### 2.2.3. Extraction of Nanocellulose

**Figure 1:**
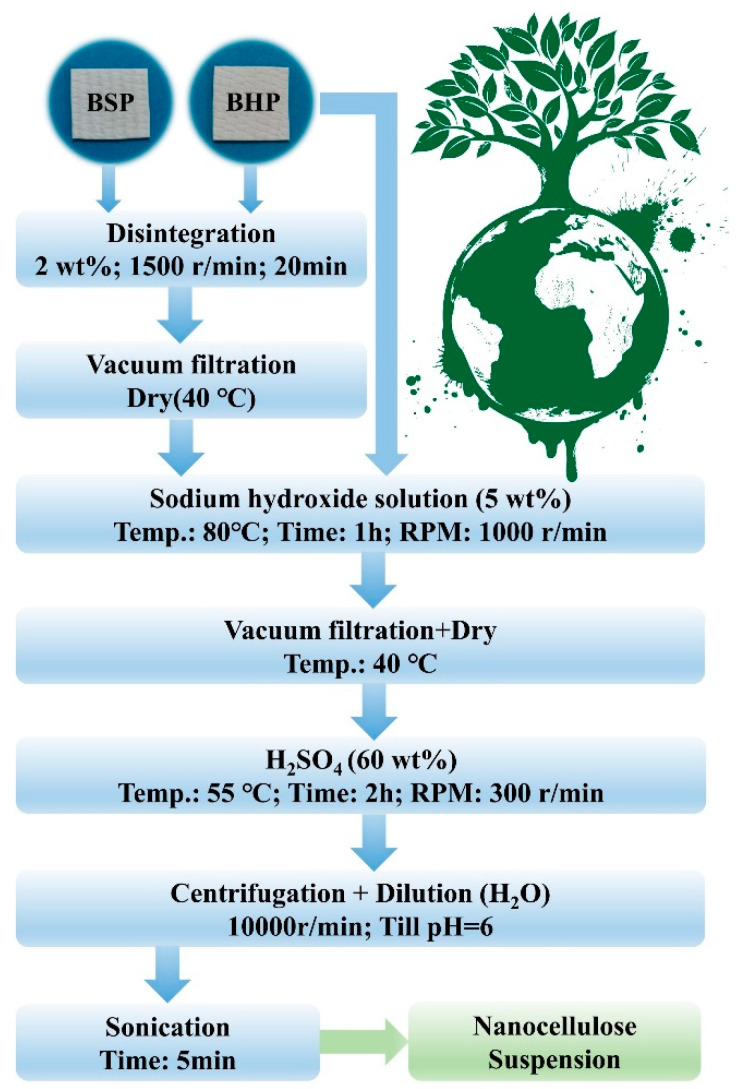
Flow chart of fabricating nanocellulose.

A total of 1.5 g dry alkali-treated pulps were added to 75 g sulfuric acid solution at the concentration of 60 wt% for hydrolysis at 55 °C for 2 h at the stirring rate of 300 r/min. All hydrolyzed nanocellulose were centrifuged for 6 min with 10,000 r/min (RCF = 10,145) and further washed with DI water respectively. This process was operated repeatedly until the pH level of the final supernatant reached approximately 6. The sediment was collected and dispersed in DI water with aid of an ultrasonic cell pulverizer for 5 min. Afterwards, nanocellulose suspensions (i.e., CBSP, CBHP, CBSP−D, and CBHP−D) were stored in fridge at 4 °C. Figure 1 presents a schematic illustration for the detailed fabrication process of nanocellulose.

#### 2.2.4. Preparation of Nanocellulose Films

Before the casting process, 0.5 wt% nanocellulose solution was sonicated for 3 min to achieve uniform dispersion, and then was poured into a plastic Petri dish, which was followed by drying at 40 °C in the air−circulating oven. Nanocellulose films were finally peeled off and stored prior to mechanical testing.

### 2.3. Characterization Techniques

#### 2.3.1. Scanning Electron Microscopy (SEM)

The morphological structures of pulp fibers were observed on a scanning electron microscope (JSM-6301F, JEOL, Tokyo, Japan) at the accelerating voltage of 20 kV. The pulps were coated with gold prior to SEM examination. The width of pulp fibers was measured by open-source Image Tool software with ten random counts to obtain the average sizes.

#### 2.3.2. Transmission Electron Microscopy (TEM)

The dispersion of nanocellulose was investigated by means of a transmission electron microscope (Talos-F 200X, FEI, Hillsboro, OR, USA) at the accelerating voltage of 200 kV. Prior to the TEM analysis, a drop of 0.1 wt% dilute nanocellulose suspension was deposited on a fresh carbon−coated copper grid, which was followed by drying at ambient temperature. Based on a similar post-processing approach via Image Tool, the average fiber length was determined from ten random measurements accordingly.

#### 2.3.3. X-ray Diffraction (XRD)

The XRD analysis of raw pulps and nanocellulose films was conducted on an X-ray diffractometer (Smartlab, Rigaku, Japan) with Cu−Ka radiation (*λ* = 1.54 Å) at an accelerating voltage of 45 kV and a current of 200 mA. The data were collected with 2*θ* ranging from 10° to 40° at a scanning rate of 10 °/min where *θ* is the diffraction angle in XRD analysis. The crystallinity index (*CrI*) of nanocellulose was calculated using the following equation [26]:

*CrI* (%) = [(*I*_200_ − *I_am_*)/*I*_200_] × 100% (1)

where *CrI* denotes relative crystallinity, *I*_200_ is the maximum intensity for both crystalline and amorphous phases in which the peak is positioned between 22.4° and 22.7° [27,28]. *I_am_* is the diffraction intensity of amorphous phase in 2*θ* range between 18° and 19° [29].

#### 2.3.4. Fourier Transform Infrared Spectroscopy (FTIR)

A small piece of sample was cut from the film prepared in Section 2.2.4 and tested directly. The infrared spectra of purified nanocellulose films (i.e., CBSP, CBHP, CBSP−D and CBHP−D) were investigated by a Fourier transform infrared spectrometer (FTIR, Agilent Cary 630 FTIR, Agilent, Palo Alto, USA) in a wave−length range of 800–4000 cm^−1^ in a transmission mode.

#### 2.3.5. Thermogravimetric Analysis (TGA)

Thermal stability of raw pulps and nanocellulose films was analyzed on a thermal gravimetric analyzer (Q500, TA Instruments, Miford Massachusetts, USA) from room temperature to 800 °C. 5 ± 0.5 mg samples were heated under N_2_ atmosphere with the purge flow rate of 60 mL/min at a heating rate of 10 °C/min.

#### 2.3.6. Mechanical Analysis

Before mechanical test, nanocellulose films were cut into a rectangular shape of 30 mm × 3 mm. A dynamic mechanical analyzer (DMA, Q800, TA, Miford Massachusetts, MA, USA) was utilized in a static mode with the preload force of 0.1 N/min in order to determine tensile properties of nanocellulose films. Three specimens for each film batch were tested to determine the average data of tensile property for reproducibility.

## 3. Results and Discussion

### 3.1. Morphology of Pulps

**Figure 2:**
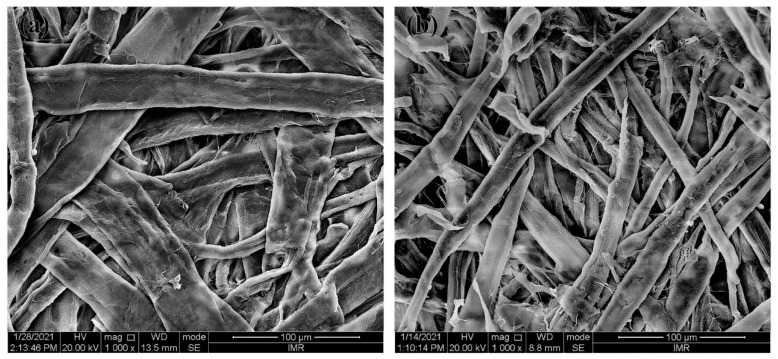
SEM images of pulps: (**a**) BSP; (**b**) BHP.

Figure 2 shows the SEM morphology of pulps. Obviously, the average widths of softwood pulp fibers (BSP = 40.59 ± 2.60 μm) are approximately two-folds as large as those of hardwood pulp fibers (BHP = 20.1 ± 0.85 μm), illustrated in Figure 2. Such a dimensional variation of pulp fibers is mainly ascribed to different species and growth regions of raw wood plants [30]. In particular, softwood fibers appear to be far longer and wider as compared with other wood fiber counterparts. Different surface morphologies of such fibers depend on the diverse types of pulps. As seen in Figure 2a, softwood fibers possess wrinkled surfaces along with a typical sign of micropores and a slight splitting on some fibers. BHP fiber surfaces shown in Figure 2b reveal furrows and cracks along the fiber longitudinal direction. It is no doubt that diverse pulp morphology and composition can be closely associated with the specific pulping process including many steps from materials preparation, pulp cooking, pulp washing to pulp bleaching and other treatments, which involve some relevant equipment such as pulp digester, vacuum drum washer, disc filter, twin roll press, single screw press, etc., [1]. After the combined physical and chemical processing treatments, lignin and hemicellulose enable to be removed from bleached fibers and many folds, grooves, and fiber splitting take place on the fiber surfaces [31,32,33].

### 3.2. Morphology of Nanocellulose

**Figure 3:**
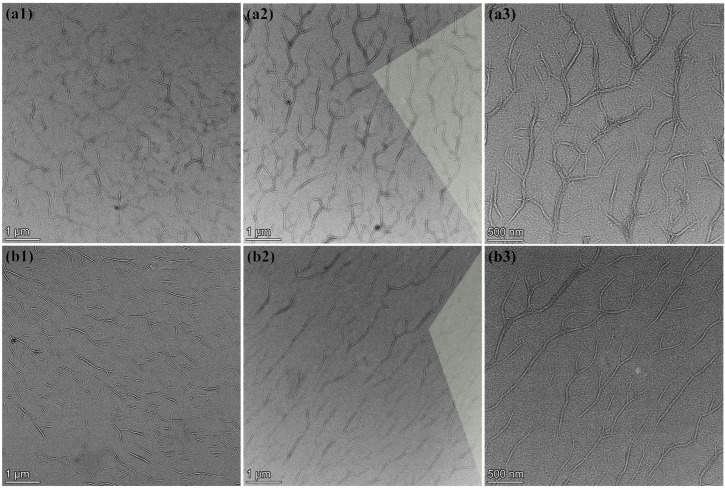
TEM images of nanocellulose: (**a1**) CBSP; (**a2**,**a3**) CBSP-D; (**b1**) CBHP; (**b2**,**b3**) CBHP-D.

TEM images of the nanocellulose are presented in Figure 3. As illustrated in Figure 3(a1) for CBSP, the nanofibers become interlaced and promiscuous, which are stacked with one another. The secondary tiny branches also exist on some primary nanofibers, resembling randomly distributed tree-branched networks. Meanwhile, such nanofibers are also demonstrated in Figure 3(a1) to be easily agglomerated owing to strong hydrogen bonding and van der Waals interaction arising in intermolecular structures of nanocellulose [34]. In comparison, CBHP is comprised of shorter primary nanofibers where more branches have been found in Figure 3(b1) to be arranged in a similar parallel direction with uniform fiber distribution and minor agglomeration. The average length of CBHP at 0.92 ± 0.21 μm appears to be non-uniform and shorter than that of CBSP at 1.26 ± 0.20 μm. As such, hardwood pulps tend to be more easily attacked by acid as opposed to softwood pulps, which is in good accordance with previous work [35]. It is believed that morphological characteristics of nanocellulose depend on the types and origins of the pulps [30].

On the other hand, relevant micrographs of CBSP−D and CBHP−D nanocellulose derived from wood pulps using pre-disintegration treatment are displayed in Figure 3(a2) and 3(b2) respectively. It is worth mentioning that average length of CBSP−D at 6.05 ± 0.54 μm has been detected to be much larger than its counterparts of CBSP without pre-disintegration treatment at 1.26 ± 0.2 μm. Corresponding 3D tree-branched network characteristic is more pronounced with a typical feature of tree growth arrangement. In order to investigate the impact of mechanical disintegration process on the morphology of nanocellulose, hardwood pulps also underwent the same treatment illustrated in Figure 3(b2). Apparently, CBHP−D exhibits the similar characteristic to CBSP−D in a tree-branched feature with longer fiber length to bear more long branches. The average length of CBHP−D at 5.36 ± 0.61 μm is significantly longer than that from CBHP at 0.92 ± 0.21 μm. It is surprising that these nanofibers are regularly distributed without typical entanglement, which is different from those rod-like cellulose nanowhiskers in presence of random distribution with a clear sign of some agglomerates. 

**Figure 4:**
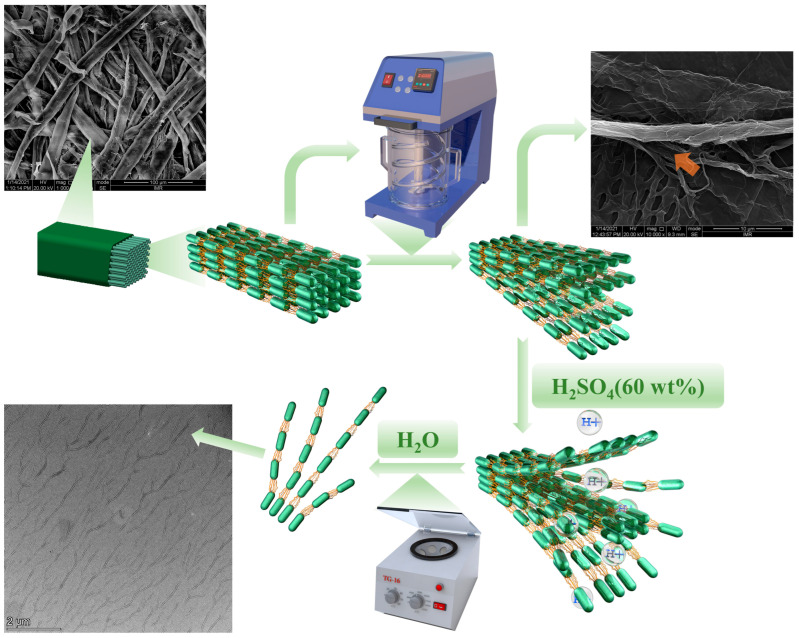
Schematic diagram of tree-branched nanocellulose (CBHP-D).

The critical impact of disintegration process on the morphology of nanocellulose is revealed in Figure 3(a2,b2). Prior to the disintegration process, the pulps were placed into water to swell, thus allowing cellulose to absorb water. Water molecules inevitably penetrate into internal areas of microfibers and hydrogen bonds are broken between microfibers, which are replaced by hydrogen bonds of microfibers and water in order to create the gaps between cellulose fibrils and microfibers. This phenomenon is believed to contribute to the disintegration process. Fiber dissociation is deemed as a disintegration process of wet fibers with the rotation of propeller at the high speed. It appears that the pulps when subjected to mild shear forces tend to become much looser while individual fibrils split along the fiber direction to a certain extent without shortening the fibers. Such shear force is much weaker than those of high-pressure homogenizer and high-speed grinding machine [21,36]. When swollen pulps were placed in the disintegrator with the propeller rotating at the high speed to induce the collision of water and pulp fibers, single-molecule water impinges on fiber surfaces and gradually enters the interior of fibers, which eventually breaks hydrogen bonds and van der Waals forces between the microfibrils from the outer to inner side. With the aid of continuous rotation, the relevant gaps between adjacent cellulose fibrils are gradually enlarged leading to typical cracks that are further split from the fibers, as well as a higher degree of fiber splitting, as shown in Figure 4 (red arrow). Such a process assists in the formation of tree-branched nanocellulose via acid hydrolysis since gap spaces of microfibrils make it easy for sulfuric groups and hydronium ions to penetrate into fibers. In general, amorphous phase of cellulose fibers is preferentially removed in acid hydrolysis, which is mostly like to be involved with two critical steps, as mentioned earlier by Spiliopoulos et al. [37]. The first step is that pulp fiber surfaces are swollen in sulfuric acid solution. In the meantime, hydronium ions attack amorphous phase of cellulose fibers, resulting in the breakage of 1, 4−β−glycosidic bonds. As sulfuric acid reaches the inner side of cellulose fibers in a layer-by-layer manner in a swelling−hydrolysis process, fiber chains are split for the formation of thinner nanocellulose fibers [38]. In the process of acid hydrolysis, undisintegrated pulps can also adopt such a hydrolysis mechanism that the breakage of hydrogen bonds and van der Waals forces are achieved by swelling from surfaces to the interior of cellulose fibers [39]. All hydronium ions solely act on the surfaces in relation to amorphous phase of cellulose microfibers, which means that only cellulose microfiber surfaces are completely exposed to highly concentrated sulfuric acid solution in order to break 1, 4−β−glycosidic bonds. Accordingly, nanocellulose with smaller sizes can be formed more easily, as evidenced by Figure 3(a1,b1). Acid hydrolysis process confirms that cellulose is hydrolyzed from outer to inner side in a layer−by−layer manner. On the contrary, disintegrated pulps undergo a different hydrolysis process. As pulp fibers tend to be much looser after the disintegration treatment, large gaps are identified between the microfibers along with the splitting of some fibrils in the fiber direction. As such, disintegrated pulps are swollen with the sulfuric acid solution, which are different from undisintegrated pulps. Additionally, the gaps between cellulose microfibers appear to be much larger than ever before. Such a phenomenon is conducive to allow hydronium ions and sulfuric groups to penetrate into adjacent cellulose microfibrils. That means pulp fibers with higher specific surface areas are exposed to sulfuric acid solution, and hydronium ions are much easier to uniformly attack amorphous phase of fibrillar surfaces and the interior simultaneously. This effect suggests that acid hydrolysis on the capacity of breaking 1, 4−β−glycosidic bonds should be reduced, which would contribute to the retention of more amorphous phase to generate long tree-branched nanocellulose, as illustrated in Figure 3(a2,b2). This finding is consistent with degree of crystallinity that will be presented in Figure 6c, indicating that CBSP−D and CBHP−D nanocelluloses have lower degree of crystallinity when extracted from disintegrated pulps. Consequently, it is not hydrolyzed from outer to inner layers of pulp fibers in a layer-by-layer manner, which is essentially different from the hydrolysis mechanism of undisintegrated pulps. 

**Figure 5:**
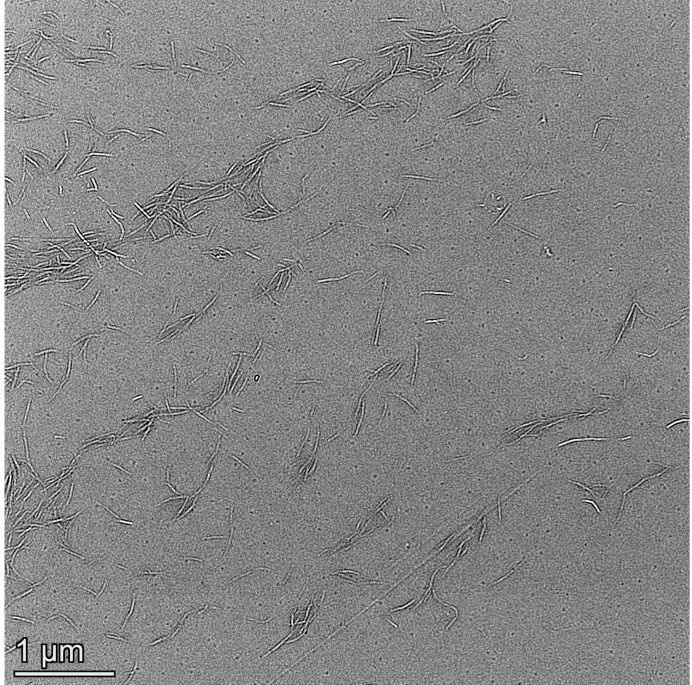
TEM image of nanocellulose rods from BHP−D under hydrolysis for 3 h.

The unique tree-branched formation of nanocellulose achieved in this study can be associated with several different reasons. The first lies in a mild acid hydrolysis condition. Tree-branched nanocellulose is believed to be an intermediate transition state from the pulps to rod−like nanocrystals, which can occur under hydrolysis at very vigorous conditions including higher acid concentration and higher temperature with the longer duration. To confirm this, hydrolysis time is further extended while maintaining the other conditions in terms of acid concentration, temperature, and stirring speed in order to obtain rod-like nanocellulose shown in Figure 5. Furthermore, the pre−disintegration process before hydrolysis becomes favorable to the formation of tree−branched nanocellulose due to the splitting of fibers. Since internal structures of fibers are loose, more cellulose microfibrils are exposed to sulfuric acid solution, which makes it easy for the acid to attack interfibrillar parts for achieving long fibers in the longitudinal direction. Overall, the pre-disintegration process plays an important role in the formation of tree−branched nanocellulose. 

### 3.3. X-ray Diffraction (XRD) Analysis

**Figure 6:**
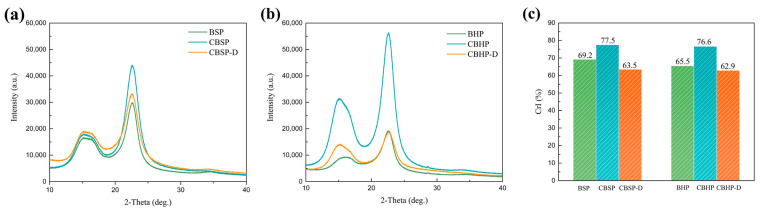
XRD patterns of different material samples: (**a**) BSP, CBSP, and CBSP-D; (**b**) BHP, CBHP, and CBHP-D; (**c**) the degree of crystallinity of material samples (BSP, CBSP, CBSP-D, BHP, CBHP, and CBHP-D).

Figure 6 depicts the diffraction patterns of raw pulps and nanocellulose. The XRD pattern of pulps exhibit typical cellulose I characteristic peaks at 2*θ* = 15.2°, 16.4°, and 22.6°, which corresponds to ($1\bar{1}$0), (110), and (200) crystal planes [27,28], as shown in Figure 6a,b, with the overlapping of ($1\bar{1}$0) and (110) reflections. After acid hydrolysis, the characteristic peaks of nanocellulose for CBSP and CBHP are similar to those of the pulps despite higher intensities taking place at 15.2° and 22.6° (Figure 6a,b). Obviously, XRD patterns of nanocellulose are attributed to those of their corresponding pulps. As shown in Figure 6a,b, the diffraction peak positions of CBSP−D and CBHP−D are consistent with those of CBSP and CBHP respectively. However, diffraction peak intensities of CBHP−D and CBSP−D can be significantly decreased possibly due to more amorphous contents.

Figure 6c displays the degree of crystallinity of nanocellulose. The degree of crystallinity of nanocellulose increases significantly after acid hydrolysis resulting from the hydrolysis of more amorphous cellulose. Conversely, the degree of crystallinity for CBSP−D and CBHP−D is significantly lower at 63.5% and 62.9% accordingly when compared with those of CBSP at 77.5% and CBHP at 76.6%. This result indicates that the combined disintegration and acid hydrolysis yield more amorphous phase observed in nanocellulose, which is in good agreement with the morphologies of CBSP−D and CBHP−D shown in Figure 3(a2,b2). 

### 3.4. Fourier Transform Infrared Spectroscopy (FTIR) 

**Figure 7:**
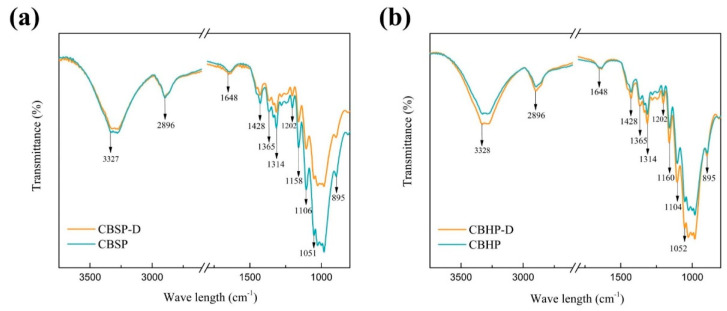
FTIR spectra of different material samples: (**a**) CBSP−D and CBSP; (**b**) CBHP−D and CBHP.

The impact of pulp species or pulp pretreatment process on FT-IR spectra of nanocellulose was investigated to provide some insights to molecular interactions involved, as illustrated in Figure 7. FTIR is used to characterize chemical structures by identifying functional groups of material samples. The peak position of nanocellulose (i.e., CBSP−D, CBSP, CBHP−D, CBHP) does not change significantly. There are broad peaks detected between 3274 and 3328 cm^−1^ and at 2896 cm^−1^ respectively, which correspond to the O−H stretching vibration and C−H bond stretching vibration in methyl and methylene groups of cellulose [34]. The characteristic peaks are assigned to 1646 cm^−1^ with respect to O−H bending of absorbed water in nanocellulose molecules [40]. The peak in the range of 1420–1430 cm^−1^ is associated with the CH_2_ scissoring motion of cellulose, but the specific band at 1428 cm^−1^ is also related to amorphous cellulose. The peaks identified at 1365 cm^−1^ and 1314 cm^−1^ correspond to the C−H bending and CH_2_ wagging respectively [41]. The characteristic peaks in range of 1170–1082 cm^−1^ are assigned to the C−O−C bond in pyranose ring skeletal of cellulose [40]. There is a peak assigned to 1052 cm^−1^ due to C−O bond stretching vibration at C6 in cellulose [34,42]. The bands at approximately 1028 cm^−1^ and 982–984 cm^−1^ are ascribed to C−O deformation and stretching at C6 in cellulose accordingly. The peak occurring at 895 cm^−1^ is correlated to C−O−C stretching β− (1−4) −glycosidic linkages [41,43,44]. The relevant peaks of nanocellulose appeared in the range of 1200–1290 cm^−1^, which usually correspond to the C−O−C bending in aryl-alkyl ether of cellulose or in aryl-alkyl ether of lignin. In fact, nanocellulose hardly contains any lignin, and characteristic peaks are absent with respect to aromatic ring vibrations in the range of 1700−1730 cm^−1^, C=C bond of benzene stretching ring at 1632 cm^−1^, and C=C bond of aromatic skeletal mode at 1613 cm^−1^ and 1450 cm^−1^ [40].

### 3.5. Thermal Stability Analysis

**Figure 8:**
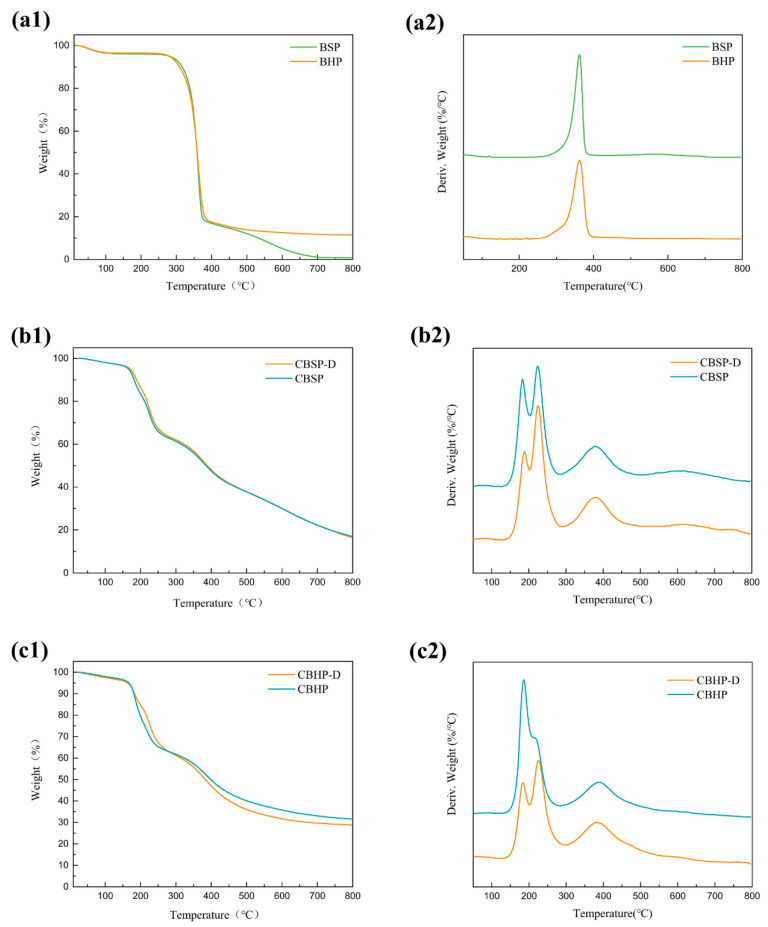
TGA diagrams of all material samples: (**a1**,**a2**): pulps; (**b1**,**b2**): CBSP−D and CBSP; (**c1**,**c2**): CBHP−D and CBHP.

As seen in Figure 8(a1), TGA diagram of the pulps demonstrates that initial small mass loss takes place at 100 °C arising from the evaporation of absorbed moisture, which is followed by hemicellulose decomposition at approximately 247 °C according to Table 1. The maximum decomposition temperature takes place at approximately 362 °C as shown in Figure 8(a2). Such a decomposition process continues until the temperature reaches 400 °C at which most cellulose materials have been pyrolyzed [45]. The mass loss rate of BHP seems very slow and remains about 11.4 wt% for the temperature level beyond 400 °C. Nonetheless, it has been detected that BSP degradation continues up to 718 °C where there is very little solid residue of 0.73 wt% left. Such results can be interpreted by charred residues or oxides turning into small-molecule gasses [46]. 

TGA diagrams of nanocellulose, depicted in Figure 8(b1,c1), present initial lower degradation temperatures when compared with those of pulps according to Table 1. This is because sulfation groups on nanocellulose surfaces could accelerate the degradation of nanocellulose [29,46]. The second degradation process of CBSP and CBHP nanocelluloses is considered as a major degradation taking place in the range of 135−290 °C, which appears to be related to bimodal mass loss mode with different maximum mass loss rates of nanocellulose, as evidenced in Figure 8(b2,c3) and Table 1. Such a phenomenon is attributed to two-step degradation of nanocellulose with sulfate groups existing at the end of crystalline cellulose and sulfated amorphous cellulose [46]. A short and stable stage is identified with respect to the degradation process of all nanocelluloses just prior to the further degradation between 290 °C and 500 °C where maximum decomposition temperature occurs at approximately 385 °C in Table 1 owing to the evident degradation of unsulfated interior crystalline phase [47]. Finally, the degradation continues to increase up to 800 °C in a relatively slow pace. On the other hand, thermal stability behaviors of CBSP-D and CBHP−D nanocelluloses are revealed in Figure 8(b1,b2), as well as 8(c1,c2) respectively, along with corresponding characteristic parameters listed in Table 1. In contrast, the maximum mass loss rate detected in the second degradation process in range of 135–290 °C differs from those of CBSP and CBHP nanocelluloses without pre-treatment. As clearly shown in Figure 8(b1,b2), the maximum mass loss rates of CBSP−D and CBSP nanocellulose have been found to occur in temperature ranges of 183−188 °C and 223−225 °C respectively. 

More importantly, the ratio of peak intensity, referred to as the latter maximum mass loss rate to the former maximum mass loss rate, is quite different, as indicated by CBSP-D = 1.47 and CBSP = 1.11 accordingly. Such a result may be associated with different size and degree of crystallinity of cellulose materials used in this study. As mentioned earlier in Figure 3(a1,a2), the average fiber length of 6.05 μm for CBSP−D is significantly larger than that of CBSP in contrast with the lower degree of crystallinity of CBSP−D as opposed to that of CBSP shown in Figure 6c. It means that CBSP−D possesses more sulfated amorphous phase than CBSP, thus resulting in changing the maximum mass loss rate of nanocellulose. Similar phenomena are demonstrated in Figure 8(c1,c2) as well. A ratio of peak intensity at 1.28 is present in CBHP−D, much higher than that of CBHP at 0.58. Such findings suggest that sulfated amorphous phase of nanocellulose is more likely to degrade at approximately 225 °C in accordance with the associated peak for second maximum mass loss rate. As such, it is quite convincing that main degradation of nanocellulose would be probably influenced by its amorphous content. 

### 3.6. Mechanical Properties of Nanocellulose Films

**Figure 9:**
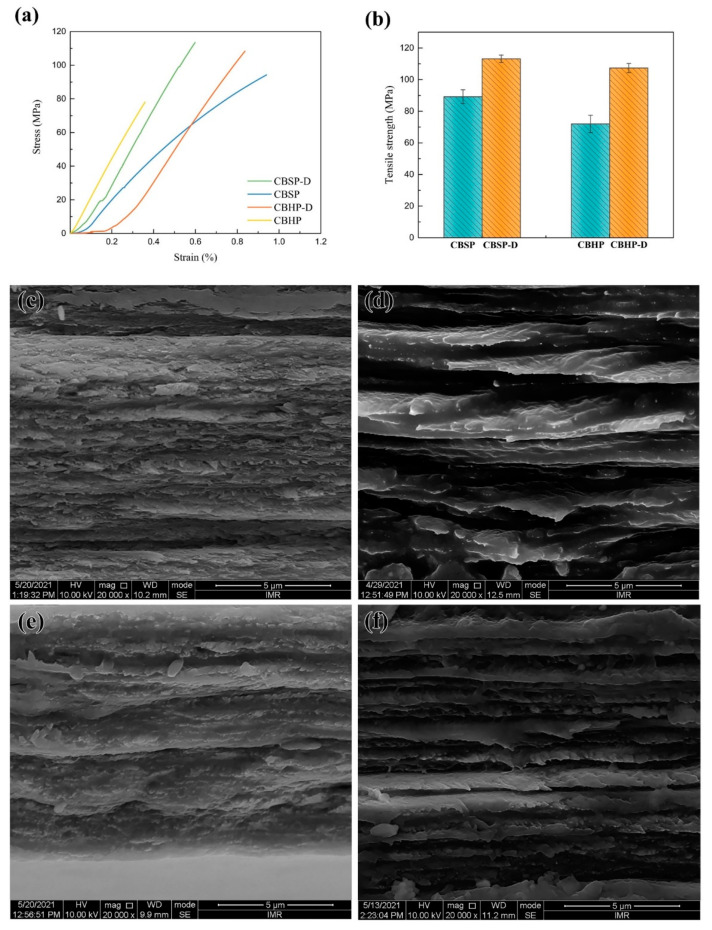
Tensile properties of nanocellulose films: (**a**) stress−strain curves; (**b**) tensile strength, as well as cross-sectional images of (**c**) CBSP, (**d**) CBSP−D, (**e**) CBHP and (**f**) CBHP−D films.

Typical stress–strain curves and fracture morphologies of different nanocellulose films are depicted in Figure 9. It is manifested that the tensile strength of CBSP films at 89.18 MPa appears to be higher than that of CBHP counterparts at 71.99 MPa, which is impacted by nanofiber morphology and size, as well as branching degree in relation to contract areas between fibers [48,49]. CBSP is derived from softwood pulps produced in Russia with typical interlaced, tangled, and interwoven structures, as evidenced by Figure 3(a1). This results in the formation of interconnected networks of multiple hydroxyl bonds in order to improve interfiber bond strength. Moreover, such networks enable to facilitate the effective stress transfer to neighboring fibers for enhancing mechanical properties of nanocellulose films [50]. 

The impact of disintegration process on the tensile strength of nanocellulose films was investigated, as shown in Figure 9. In comparison, CBSP−D films possess much higher tensile strength of 113.47 MPa when compared with CBSP counterparts due to unique morphological characteristics of CBSP−D presented in Figure 3(a1) where their networks resemble blood vessels to yield a higher degree of continuous contact areas for interfiber bonding effect. Another plausible reason lies in the abundance of hydroxyl groups on nanocellulose surfaces, as indicated by four hydrogen bonds between fibers per unit cell [48,49]. It may imply that more hydrogen bonds can be generated between these long tree-like nanocellulose fibers. Figure 9d reveals a more densely packed layered structure that is perpendicular to the film direction by using strong hydrogen bonds for CBSP−D. Non-continuous contact areas of CBSP diminish because of shorter fiber length leading to more interrupted sites per unit area shown in Figure 9c in order to delay local stress transfer. Figure 9e,f displays a similar trend in relation to tensile strength and fracture morphology where the strength levels of 108.31 and 71.99 MPa have been achieved for CBHP−D and CBHP accordingly. 

In particular, tensile strengths of CBSP−D and CBHP−D films are compared with nanocellulose films in previous studies in Table 2. The mechanical properties of nanocellulose films possess a wide variations depending on raw materials, sizes, morphologies, and degree of crystallinity of the fibers. The tensile strength of nanocellulose film was reported to be 150 MPa for the specific fibers (fiber diameter of 9.1 nm and aspect ratio of 12.6), which were extracted from bleached wood pulps by sulfuric acid hydrolysis [51]. However, some nanocellulose film had a relatively low strength of 70 MPa (standard deviation, SD = 29 MPa) though the nanocellulose exhibited fiber diameter of 7.9 nm (SD = 2.5 nm) and aspect ratio of 22 (SD = 13) [52] owing to fiber inhomogeneity. The tensile strengths of CNF/CMF films show the same variations as those of CNC/CNW films. A superior tensile strength of 197 MPa was achieved for CNF films. The fibers were obtained by TEMPO-oxidation and high-pressure homogenization treatments along with a carboxylate content of 1.8 mmol/g [53]. It is well−known that bacterial cellulose films can possess a high strength over 250 MPa due to high purity and uniformity of cellulose. Those nanocelluloses under pre-treatment of disintegration prior to acid hydrolysis have longer fibers with more branches in order to benefit the increase in tensile strength. 

## 4. Summary

In this study, cellulose nanofibers were produced from various wood pulps by conventional acid hydrolysis methods. CBSP and CBHP nanocelluloses possess a unique tree-branched structure. The fiber length of CBSP at 1.26 μm derived from softwood pulps is greater than that of CBHP at 0.92 μm. Thermal stabilities of all nanocellulose are consistently poor as opposed to those of wood pulps. BSP and BHP wood pulps under the disintegration treatment lead to unique morphological characteristics comprising self-organized 3D networks resembling blood vessels for CBSP-D in size of 6.05 μm, as well as long tree-branched fiber structures with the parallel orientation for CBHP−D at 5.36 μm. The addition of amorphous phase results in decreasing the degree of crystallinity of CBSP−D at 63.5% and CBHP−D at 62.9%. Tensile strengths of CBSP−D and CBHP−D are superior to those of CBSP and CBHP in mechanical properties of nanocellulose films.

This study clearly demonstrates a tree-branched morphology of nanocellulose via sulfuric hydrolysis methods under mild stirring condition, which is not achieved by conventional acid hydrolysis methods. It is believed that nanocellulose morphology is highly associated with the sources of raw cellulose fibers and hydrolysis conditions. Currently, it is speculated that this particular formation is an intermediate state from cellulose microfibers to cellulose nanowhiskers. It has been proved that these tree-branched nanofibers will be disintegrated into nanowhiskers once the hydrolysis becomes intense. The in-depth investigation on the future applications is of great importance for developing more advanced nanocellulose materials. The strong interactions among these tree−branched cellulose nanofibers show their great potentials as reinforcing fillers, transparent film in packaging and optoelectronic, Picker emulsion stabilizer will be advantageous over the rod-like cellulose nanowhiskers. However, the relationship between raw materials/processing parameters and morphology of nanocellulose should be clarified to investigate the mechanism for the formation of various morphologies of nanocellulose. Currently, our understanding on these tree-branched nanocellulose is quite limited. A lot of work should be done to further explore the particular properties and usage of the tree−branched nanocellulose. Overall, the successful fabrication of tree-branched nanocellulose is anticipated to offer widespread applications and advances in cellulose manufacturing technologies at the industrial level.

## Figures and Tables

**Table 1:** Initial degradation temperatures of material samples and maximum mass loss rates at diverse temperature ranges.

Sample	Initial Degradation Temperature (°C)	Maximum Decomposition Temperature T_max_(°C)	Maximum Mass Loss Rate (%/°C)
BSP	251	362	2.51
BHP	247	362.1	1.93
CBSP	135	183.0	0.43
		223.2	0.48
		379.4	0.18
CBHP	135	185.9	0.62
		214.9	0.36
		386.6	0.17
CBSP-D	137	187.9	0.36
		224.8	0.53
		379.4	0.19
CBHP-D	135	183.5	0.36
		225.6	0.46
		384.4	0.19

**Table 2:** Tensile properties of nanocellulose films.

Nanocellulose Film	Tensile Strength (MPa)	References
CNCs/CNWs	70–150	[51,52]
CNFs/CMFs	48.9–197	[51,53,54,55]
BC	256–265	[56,57]
CBSP-D I	113.47	this study
CBHP-D	108.31	this study

## Data Availability

The data presented in this study are available upon request from the corresponding authors.
